# Developing quality criteria for patient‐directed knowledge tools related to clinical practice guidelines. A development and consensus study

**DOI:** 10.1111/hex.12843

**Published:** 2018-11-11

**Authors:** Trudy van der Weijden, Dunja Dreesens, Marjan J. Faber, Nanne Bos, Ton Drenthen, Ingrid Maas, Sonja Kersten, Uriëll Malanda, Sander van der Scheur, Heleen Post, Anouk Knops

**Affiliations:** ^1^ MUMC+ CAPRHI Maastricht The Netherlands; ^2^ Radboudumc IQ Healthcare Nijmegen The Netherlands; ^3^ Netherlands Institute for Health Services Research NIVEL Utrecht The Netherlands; ^4^ Dutch College of General Practitioners Utrecht The Netherlands; ^5^ Dutch Association of Medical Specialists Utrecht The Netherlands; ^6^ Dutch Nurses’ Association Utrecht The Netherlands; ^7^ Health Care Institute of the Netherlands Diemen The Netherlands; ^8^ Dutch Federation of Patients’ Organisations Utrecht The Netherlands

**Keywords:** clinical practice guidelines, patient decision aids, patient information, patient involvement, patient participation, patient versions of guidelines, quality standards, shared decision making

## Abstract

**Background:**

Patient‐directed knowledge tools such as patient versions of guidelines and patient decision aids are increasingly developed to facilitate shared decision making. In this paper, we report how consensus was reached within the Netherlands on quality criteria for development, content and governance of these tools.

**Method:**

A 12‐month development and consensus study. The consortium worked on four work packages: (a) reviewing existing criteria; (b) drafting the quality criteria; (c) safe‐guarding the acceptability and feasibility of the draft criteria by participatory research in on‐going tool development projects; and (d) gaining formal support from national stakeholders on the quality criteria.

**Results:**

We reached consensus on a 8‐step guidance; describing minimal quality criteria for (a) the team composition; (b) setting the scope; (c) identifying needs; (d) the content and format; (e) testing the draft; (f) finalizing and approval; (g) dissemination and application, and (h) ownership and revision. The participants of the on‐going tool development projects were positive about the quality criteria in general, but divided as to the degree of detail. Whereas some expressed a clear desire for procedural standards, others felt that it would be sufficient to provide only general directions. Despite the different views as to the degree of detail, consensus was reached in three stakeholder meetings.

**Discussion:**

We successfully collaborated with all stakeholders and achieved formal support from national stakeholders on a set of minimum criteria for the development process, content and governance of patient‐directed knowledge tools.

## 1 INTRODUCTION

The knowledge in health care is expanding daily—so that keeping up with knowledge is a challenge.^1^ The development of knowledge tools is intended to support clinicians to keep pace and to improve their decision making. Many knowledge tools such as clinical practice guidelines, protocols or clinical pathways have been developed over the years.^2^ With the increasing call for a patient revolution,^3^ further tool types have been added to the mix, including patient decision aids. A key source of information for patient‐directed knowledge tools is clinical practice guidelines. Clinical practice guidelines summarize research evidence systematically and provide recommendations on a specific clinical topic.^4^ Nowadays, the GRADE approach is used as a framework to rate the quality of the evidence and to assess the strength of the recommendations taking into account the balance between benefits and harms, resource use and feasibility considerations. The GRADE method also recognizes the collective patient perspectives;^5^ the strength of the recommendations is also affected by the patients’ appreciation of advantages and level of acceptability of disadvantages of the intervention, such as side‐effects and treatment burden. Worldwide, patients and patient representatives increasingly take part in the development of guideline recommendations.^6^

Next to patient participation on a collective level, efforts are made to adapt or enrich guidelines so as to facilitate patient participation on an individual level, in clinical decision making.^7^ Patient participation is especially important in case of preference‐sensitive decisions where multiple options exist or where the benefits and harms of the intervention may be assigned a different weight by different patients.^8^ Illustrative examples to facilitate patient participation in clinical decision making are patient versions of a guideline such as a lay summary, or patient decision aids for specific preference‐sensitive decisions attached to the guideline document. Some guidance for the content of lay summaries of guidelines is provided by the Guidelines International Network.^9^ In 2006, standards were formulated for the content of patient decision aids by the International Patient Decision Aids Standards (IPDAS) group, a multistakeholder process that led to a self‐assessment checklist.^10^ Further work led to a measure IPDASi^11^ and a set of criteria that should be met to achieve a “minimum” acceptable standard.^12^ Guideline developers are experimenting to derive the information for patient decision aids—evidence on benefits and harms of interventions and on patient considerations and patient preferences—directly from the guideline.^13^

Much is also happening with respect to development of patient‐centred knowledge tools such as patient versions of a guideline or patient decision aids in the Netherlands, on various sides of the care equation.^14^ Patient organizations are gaining a more accurate picture of the information needs that these tools must satisfy. Professional and scientific associations feel a responsibility to ensure the accuracy and effectiveness of medical information supplied via such tools. Web and tool designers continue to introduce ever more user‐interface friendly tools.

For this study, we used the following definition: a patient‐directed knowledge tool synthesizes and distils the highest quality knowledge and research, is aimed directly at the patient (and next of kin), with the goal to engage patients in dialogue or deliberation during a clinical encounter or to support and/or improve patient decision making which may or may not take place during a clinical encounter.^15^ Yet these patient‐centred knowledge tools are subject to a multitude of varying definitions and criteria, especially regarding the patient versions of guidelines, and the development process.^16^, ^17^, ^18^, ^19^, ^20^, ^21^, ^22^ As a result, it is difficult for parties to distinguish what is truly important from what is not or what type of patient‐directed knowledge tool is in fact the correct means to achieve the stated purpose. In addition to quality criteria, the need for national governance is also felt strongly, as many initiatives by patient organizations and professional bodies to develop patient‐directed knowledge tools exist side by side. This situation has resulted in an uncoordinated, partly overlapping mixture of publicly and privately/commercially available patient decision aids.^23^ Moreover, some of the patient decision aids do not seem to follow the rigid and multistakeholder methods to review the evidence base, as is common in clinical practice guidelines.

A guidance for the development of reliable patient versions of guidelines and patient decision aids can serve to integrate all existing knowledge and previously developed expertise, allowing the stakeholders to work together more effectively and more efficiently. The purpose of such a guidance is to promote the development of high‐quality, reliable and publicly available patient‐directed knowledge tools, which will contribute to achieving properly informed patients and shared decision making.

Initiated by the National Health Care Institute of the Netherlands, a consortium of healthcare stakeholders started to develop such *guidance*, supported by academic researchers. Apart from validity, feasibility was important given the conflict between the aim of high‐quality knowledge tools and the limited resources to develop such tools. The purpose of this article is twofold. First, we describe the methods used for arriving at the *guidance* as an illustrative example of how formal support from national stakeholders can be reached. Second, we present the list (*guidance*) of minimum quality criteria for the development, content and governance of patient information on guidelines and patient decision aids, as well as the way in which these tools can be connected to the clinical practice guidelines.

## 2 METHODS

The National Health Care Institute of the Netherlands initiated and granted this 12‐month development study that was composed of a literature review, a feasibility check and a consensus procedure. We took the position that we needed various types of input and processes to ensure a successful consensus process. To this end, we designed a consortium (see first four authors and last two authors) that worked together continuously in an iterative process using cross‐fertilization, without being hindered by hierarchy. A representative of the Dutch Federation of Patients’ Organisations (last author) was co‐leading the project with the first author.

In a 12‐month project that started in October 2015, we worked on the basis of four work packages (WPs): (WP1) Radboud University (MF) reviewed existing criteria in the literature, synthesizing evidence and best practices; (WP2) Maastricht University (TvdW, DD), the coordinating group, developed the drafts of the guidance; (WP3) NIVEL (NB) safeguarded the acceptability and feasibility of the draft criteria by gathering experiences with the draft guidance from knowledge tool developers; (WP4) the Dutch Federation of Patients’ Organisations (AK) organized the consensus procedure aiming to support the guidance by national stakeholders.

(WP1) We searched for formal criteria and methodologies in the scientific literature, in policy reports, and on websites by developers of guidelines and patient decision aids. The search strings that we used to explore PubMed are described in Table 1, as are the websites to search for the grey literature. This inventory supplied the basis for the first draft of the guidance. One of the researchers of WP1 made a first selection of the search based on title and abstract, and excluded references clearly not fulfilling the inclusion criteria. All full‐text versions that resulted from this first selection were downloaded and assessed along the pre‐set inclusion and exclusion criteria. In case of doubt, a second researcher was consulted to reach consensus on inclusion or exclusion.

**Table 1:** Search strings used to explore PubMed and websites used to search for grey literature

Search strings
Patient information based on guidelines
(((method*[Title/Abstract] OR approach*[Title/Abstract] OR framework[Title/Abstract] OR develop*[Title/Abstract] OR creat*[Title/Abstract])) AND (“patient version*”[Title/Abstract] OR “information for the public”[Title/Abstract] OR “public information”[Title/Abstract] OR “patient booklet*”[Title/Abstract] OR booklet*[Title/Abstract])) AND (“clinical practice guideline*” OR “Practice Guidelines as Topic”[Mesh] OR “quality standard*”) (“Practice Guidelines as Topic”[Mesh] OR “Practice Guideline” [Publication Type] OR guideline*) AND “patient version” AND develop*
Patient decision aids
“Decision Support Techniques”[Majr:NoExp] AND (method*[tiab] OR approach*[tiab] OR framework[tiab] OR develop*[tiab] OR creat*[tiab]) AND (“Patient Satisfaction”[Mesh] OR “Patient Participation”[Mesh] OR “Patient‐Centered Care”[Mesh]))
Websites
Dutch Knowledge Institute of Medical Specialists Netherlands Comprehensive Cancer Organisation Guidelines International Network, Patient and Public Involvement working group UK National Institute for Health and Care Excellence (NICE) German Ärztliches Zentrum für Qualität in der Medizin (ÄZQ) Finnish Duodecim Australian National Health and Medical Research Council (NHMRC) USA Oncoline Kaiser USA Agency for Healthcare Research and Quality (AHRQ) Canadian Task Force on Preventive Health Care (CTFPHC) IPDAS working Group www.ipdas.ohri.ca Patient Decision Aids Research Group https://decisionaid.ohri.ca The Preference Laboratory http://optiongrid.org/option-grids/about-the-grids Mayo Clinic for shared decision making http://shareddecisions.mayoclinic.org DECIDE research Group www.decide-collaboration.eu Joanna Briggs Institute University of Adelaide http://joannabriggs.org

Inclusion criteria for literature on patient versions of guidelines:

- The paper describes the development process of a patient version or lay summary of a clinical practice guideline.
- Explicit description of the methods used (be it short or extensive) for development.
- English or Dutch language.

Exclusion criteria:

- Papers describing only the process of patient participation in the development of a clinical practice guideline.

Inclusion criteria for literature on patient decision aids:

- The title reports the term “development” or “design.”
- The abstract reports the development of a patient decision aid as the aim of the paper.
- Description of the development process of disease‐specific or generic decision aids.
- Explicit description of the methods used (be it short or extensive) for development.
- English or Dutch language.

Exclusion criteria:

- Papers describing the development of tools that stretch further than patient decision aids (eg, social support and self‐management).
- Papers describing the development of tools on other decisions than medical decisions.

(WP2) The project was coordinated via monthly meetings of all WP leaders, complemented by numerous one‐to‐one contacts. We used definitions of the patient‐directed knowledge tools that were recently formulated in another Dutch national consensus procedure; see Box 1.^24^, ^25^ The findings from the literature review were used to draft the first set of the minimal quality criteria for development, content and governance of patient‐directed knowledge tools. The findings of the feasibility checks (WP3) and consensus meetings (WP4) were used to write the second and third draft of the guidance.

Box 1 **The Dutch definitions of the patient‐directed knowledge tools^24^, ^25^**

1 *Patient information based on a guideline* (=patient version of a guideline): Explanation of a specific condition or (health) care issue based on a guideline; made available to patients and their next of kin; provides information on available care choices and the care that they can expect from the care process.

*Summary of guideline*: Concise overview of the guideline, providing main conclusions and recommendations in clear and simple language; can be applied in practice independently from the guideline; intended for both care providers and patients.

*Patient decision aid (PDA)*: Auxiliary information and answers to frequently asked questions for patients when choosing, with their care providers, from different options—including the option to forgo care—in a specific area such as diagnostics, treatment, screening, counselling or aftercare; discusses the possible outcomes and effects of each option—desirable or otherwise—and their likelihood of occurring; helps patients to weigh up their options based on their own values, standards and personal circumstances.

(WP3) The first and second draft guidance was presented for a critical assessment of its feasibility to the project leaders of nine working groups tasked with the development of patient versions of guidelines or patient decision aids along clinical practice guidelines. These working groups were at that time in various phases of their development projects. Five projects focused on developing patient information based on guidelines, for example for patients with inflammatory bowel disease. Four projects focused on developing patient decision aids for specific recommendations, for example in the care for orthopaedic patients. For the third draft of the guidance, we did not only seek for critical assessment by the project leader, but we also asked the project leader to actually apply (part of) the guidance steps in their working groups and to report about their experiences. Four of these nine on‐going projects were further analysed by means of outreach visits and participatory observations of working group meetings. Finally, the last draft and the experiences were fed back to each project leader in individual semi‐structured qualitative interviews. The interviews were audiotaped, transcribed and analysed with thematic content analysis.^26^

(WP4) The draft versions of the guidance were discussed in three invitational meetings. We purposefully sampled the participants for the first two meetings to guarantee continuity in the process by inviting a core group for both meetings. While we planned the input from academic experts in the first meeting, the profile of participants gradually shifted to stakeholders representing end‐users only in the last meeting.

First, a 2‐hour expert meeting was held in March 2016 aimed at collecting the experts’ suggestions, for which 43 stakeholders representing patients, care providers, researchers, web and tool designers and healthcare insurers were invited. Second, a 2‐hour meeting was held in June 2016, for which 29 stakeholders representing patients, care providers, knowledge institutions, healthcare insurers and the government were invited to question their support to the draft version of the guidance. Third and finally, a 90‐minute consensus meeting was held in September 2016, for which only the formal representatives of patients, healthcare providers and healthcare insurers were invited in order to gain formal support.

## 3 RESULTS

### 3.1 How did we arrive at the guidance?

#### 3.1.1 (WP1) Inventory of existing methods and criteria in scientific and grey literature

We found 51 hits in PubMed, of which four studies were included that describe criteria for developing patient versions of guidelines (see Appendix S1). The grey literature revealed many websites publishing patient versions of guidelines, but information on how these knowledge tools were developed was scarce. Detailed descriptions were found, however, in the Guidelines International Network “G‐I‐N Public toolkit on patient and public involvement in guidelines.” For developing patient decision aids, we found 385 hits in PubMed, of which 24 studies were included; 10 more relevant publications were added by the experts in the project group (see Appendix S1). In addition, the websites revealed rich data on what exactly patient decision aids are and how they should be developed.

The criteria for the content of patient decision aids were mostly based on empirical data,^27^ while such data were more or less absent for the content of patient versions of guidelines. IPDAS criteria (*ipdas.ohri.ca*) enjoy broad support where criteria for the content of decision aids are concerned, due to their substantiation by means of systematic consensus methodology.

#### 3.1.2 (WP3) Feasibility assessment in on‐going development projects

While reactions to the ordering of the development steps in the draft guidance were unanimously positive, the project leaders were extremely divided as to the degree of detail when it came to the instructions *within* the steps of the guidance, such as how best to map the patient perspective in the scoping and needs assessment phase. Whereas some project leaders expressed a clear desire for procedural standards (*it should be clear at all times who does what and when*), others felt that it would be sufficient for the instructions in each step to provide only general direction. Concerning the other issues raised, we report those most frequently mentioned:

- Deviating from the linear ordering of the guidance should be possible. For example, the guideline working group may be no longer active, while the patient‐directed tool is urgently needed.
- Language and jargon used in the guidance were often found to be too academic.
- The amount and complexity of the work to map the patients’ perspective in the scoping and needs assessment phase, for example by organising a focus group or a questionnaire survey, were often underestimated. Due to limitations in resources and the high workload, work should not be done twice, in the guideline working group and in the patient tool development group. Moreover, the required minimum number of two patients in the team—as was prescribed in the earlier drafts—was a concern, as well as the mandatory inclusion of a representative of the guideline working group.
- Formal authorization of the tool was not regarded necessary by all stakeholders, with the argument that the guideline was already approved.
- All project leaders plead for a central portal to host the patient‐directed knowledge tools, supported by a national party taking care of the governance of the tools.

#### 3.1.3 (WP4) The consensus meetings

For the first meeting, 28 out of 43 invited experts were present. When asked to mark the most important sections of the guidance, experts prioritized the following issues: chose the right type of knowledge tool for the aim it pursues; use the guideline (recommendation) itself as the most important source of information for the knowledge tool; determine who will become the owner of the knowledge tool; make the knowledge tool easily accessible and free to use; and organize authorization by the healthcare professional organization(s) as well as the patients’ organization(s).

For the second meeting, 21 out of 29 invited were present. All stakeholders were well represented. In general, they expressed a positive attitude towards the guidance although two critical remarks were made. Firstly, multiple stakeholders emphasized to widen the scope of the guidance so that patient‐directed knowledge tools can also be developed on topics that are not covered by clinical practice guidelines, especially patient organizations claimed that the information needs of patients should determine the content of patient‐directed knowledge tools, as opposed to only following the existing guideline recommendations. Secondly, the nursing organization criticized the language of the guidance being too scientific and too much loaded with medical jargon.

The third meeting was attended by formal representatives of all parties except for the Dutch Association of Insurers, which formally declined while giving blind consent to the guidance as a token of trust in the representatives of the patients and providers. Therefore, the final meeting was attended by four participants, representing the Dutch Federation of Patients’ Organisations (HP), the Dutch College of General Practitioners (TD), the Dutch Association of Medical Specialists (IM) and the Dutch Nurses’ Association (SK). They expressed their positive intentions with regard to supporting the guidance, but only after the following issues were clarified: the minimum criteria should clearly be listed separately from the additional suggestions; developers of patient‐directed knowledge tools should be encouraged to use the guidance according to the comply or explain principle; and authorization of patient‐related knowledge tools should be done on a process level and not on the level of authorizing the content of the tools, as content was already authorized in the final phase of the guideline development process.

In retrospect, it can be observed that the quest for clear and outspoken procedural standards that was verbalized by some project leaders in WP3 was strongly echoed in the first meeting but that it faded away in the second meeting, while only crude instructions for each step were regarded sufficient in the third and final meeting.

### 3.2 The guidance

WP1 provided rich data for formulating eight distinct development steps in the guidance (Table 2). The final guidance consists of three components: (a) recommendations for which type of knowledge tool (such as a lay summary or decision aid) best fits the objectives of the development group; (b) minimum criteria for the eight development steps, content and governance of each tool (Box 2); and (c) supplemental, detailed and concrete suggestions for each step in a second layer of information (14 pages in total, not presented, available on request). Developers deviating from these minimum criteria would have to provide a rationale for why a criterion does not apply (“comply or explain”). The steps need not always be followed in linear fashion, as the guidance establishes the criteria for an effective development process rather than laying out a strictly prescribed series of ordered steps. In the event that the development of the knowledge tool (patient information on a guideline or a patient decision aid based on a specific guideline recommendation) is part of a guideline project, the development team will ideally be commissioned by the guideline working group itself.

**Table 2:** The similarities and differences between the eight development steps for (a) patient information on a guideline and (b) a patient decision aid (PDA) connected to specific guideline recommendation(s)

Typical of patient information on a guideline(s)	Development steps	Typical of PDA connected to specific recommendation(s)
	1 *TEAM* Chose members and define tasks	
Provides an overview of the entire guideline (module)	2 *SCOPE* Establish provisional scope Create inventory of existing versions	Concerns one or several specific recommendations.
Not a one‐to‐one application of guideline. Information needs may also differ from those mentioned in the guideline	3 *NEEDS* Identify information needs	Establish attributes for consideration in decision making. Needs of care providers as well.
Purposeful selection of guideline recommendations	4 *CONTENT* Content and form	International criteria are in place; IPDAS (Int. Pat. Dec. Aids standards)
	5 *TEST* Testing the concept	
	6 *FINALIZING* Finalising and obtaining approval	
	7 *DISSEMINATION* Dissemination and application	
	8 *OWNERSHIP* Management and revision

Box 2 **The guidance. A brief description of each step for the development of patient information on a guideline or a patient decision aid (PDA) connected to specific guideline recommendation(s)**

1 1. *TEAM* The team composition is discussed with the relevant patients’ and professional associations. The team has an independent chair and a process support member/secretary, along with at least one patient(‐representative) with first‐hand experience (acquired by the patients’ organization). Membership of the team is approved based on written Declarations of Interests. An editor with experience in writing copy for a non‐expert audience will be involved in the team. If the development of the tool is part of a guideline project, the team will (ideally) be commissioned by the guideline working group itself, which has budgeted the developmental work.

2. *SCOPE* The team checks the availability of existing tools and establishes the objective, the target group and the rough form of the tool.

*Patient information*: Determine where the guideline is failing to meet patients’ information needs. After all, guidelines for practice are typically drawn up from the perspective of the care provider. Whenever possible, address the major underlying questions patients have about the guideline, as well as the key recommendations.

*PDA*: Select one or more recommendations from the guideline that have to do with the decision at hand and that are preference‐sensitive in nature.

3. *NEEDS* There are multiple ways to identify the needs of patients: a review of the literature, and/or additional qualitative or quantitative methodologies for collecting data, such as focus groups or questionnaires.

*Patient information*: Concerns any additional needs that have not yet been elaborated during the guideline development, for example with regard to multimorbidity, ethnic minorities, alternative interventions and self‐management.

*PDA*: Involves questions the patients and their proxies may have when faced with taking a specific decision. Which needs, preferences and attributes influence a given patient's decision making? This might involve information needs and psychosocial needs, along with important strategies for self‐management in connection with the illness or condition, and should also include the variations between patients.

4. *CONTENT* For both type of tools describe:

- The target group and medical condition/symptom/healthcare topic.
- The guideline(s) serving as the basis (in part) for the creation of the information on evidence, etc.
- The source of funding, who has ownership, year of publication and expiry date (if applicable).
- The interests of each member of the development group *(conflict of interest)*.

*Patient information*: Describe the guideline recommendations on which patients would want to be informed in terms that a layperson can understand. Mention frequently used examples of professional jargon so that patients can become familiar with them. The patient information will additionally indicate the following aspects:

- Point out where aspects have consciously been omitted and/or emphasis has intentionally shifted (if applicable), as compared to the guideline.

*PDA*: Describe the situation/decision at hand and the relevant recommendation(s) from the guideline, in terms that a layperson would understand. The PDA will describe the following aspects (at minimum):

- An explanation that the patient has a choice; that he/she is facing a preference‐sensitive decision.
- A description of the medical/care options, including the option to wait and see (if applicable) and an explanation of the procedure for each medical/care intervention.
- The desired and undesired outcomes (side‐effects) of the medical or care options, and the burden of treatment.
- The likelihood and risks of the outcomes, expressed as numeric data with equal denominator of population in natural frequencies and an identical length of time; preferably displayed in population diagrams; framed both positively and negatively (chances of both survival and fatality, for example); and in the case of risk reduction presented, at minimum, in terms of absolute (and potentially relative) risk reduction.
- An evidence table in which the medical/care options are summarized and compared in terms of a few key aspects.
- Ensure explicit mention of the attributes found in step 3 that are important for patients to keep in mind as they consider their options and elicit their values. These attributes must contribute to the key aspects described in the evidence table.

5. *TEST* The development team will present the draft to the relevant professional, scientific and patients’ associations for the purpose of obtaining feedback. The parties will assess whether the patient perspective is sufficiently reflected, ensure understanding of people with low literacy, and if the medical content is accurate. If the guideline working group is still active, the draft will be presented to that group for feedback as well.

6. *FINALIZING* The development team establishes the final knowledge tool and presents it to the relevant professional, scientific and patients’ associations for approval. This regards approval at the process level, that is concerning the creation of the knowledge tool. Ownership is determined and formally established.

7. *DISSEMINATION* The tool will ideally be submitted to the National Health Care Institute as a section of the relevant guideline(s). The knowledge tool becomes accessible to the public and is preferably made available at a central location, including points for attention to facilitate the actual application/implementation in healthcare practice.

8. *OWNERSHIP* The owner(s) of the knowledge tool will manage the tool and determine when the information is due to be revised: in any case when the expiry date has been reached. Ideally, the need for revision of the tool will be considered when the guideline as a whole is revised.

For patient versions of guidelines, the patients’ information needs together with the subject of the guideline will form the basis for the content of the patient version; the guideline itself should be the most important source of information. Rather than assuming just one guideline as a starting point, this situation might mean that multiple guidelines will need to be integrated and translated into a single patient information document, as this process will more effectively address the desires and perceptions of the target group. Or, alternatively, it might mean that only a limited number of guideline modules will be applied in creating patient information documents. A need to develop one or more patient decision aids is especially indicated when one or more of the guideline's key recommendations are preference‐sensitive in nature.

## 4 DISCUSSION

We successfully collaborated as a consortium of researchers and end‐user representatives, with patient participation achieved at the highest level of involvement, to achieve formal support from national stakeholders on a set of minimum criteria for the development process, content and governance of patient‐directed knowledge tools related to clinical practice guidelines. What we provide is not a detailed “recipe” for development but rather a series of recommendations based on the “state of the art” and feasibility considerations.

A number of potential limitations should be mentioned. Our project was explicitly embedded in the guideline context, we did, for example not include patient versions of systematic reviews. The guideline context may be a limiting context for developing patient‐directed knowledge tools. As the starting point of a clinical practice guideline is predominantly the clinicians’ perspective, important issues for patients may not be covered in the guideline. The assignment from the National Health Care Institute was aimed specifically at guidelines in the context of *curative* health care. While the literature is unclear in this regard, it is possible that the content of the guidance might have been different had representatives from public health, long‐term and palliative health care been included. One strength of this project is the systematic approach and involvement of all national stakeholders, from patients to policymakers, with patient representatives in a co‐leading role. We believe that the involvement of all stakeholders from the writing phase of the project proposal contributed to the successful collaboration. Another strength of the project is that the guidance was developed with prospective feasibility checks parallel to the nine on‐going development projects.

The relevance of patient‐directed knowledge tools being publicly available has also been acknowledged in the UK, with one of the main institutions developing guidelines committed to develop patient decision aids based on clinical guidelines.^28^ The relevance of this process was recently underpinned by empirical evidence in the Netherlands. The Dutch College of General Practitioners launched a non‐commercial public website in March 2012 that provides easy access to patient versions of guidelines. Since its launch, the website has grown to become one of the most visited Dutch healthcare websites. Healthcare usage in primary care seems to have decreased by 12% after the launch of the website.^29^

We expect the criteria to evolve over time as they are further tested through developers using patient versions of guidelines and patient decision aids, as well as by adding new tools to the guidance. The next challenge will be the effective implementation of the guidance as a further step towards ensuring the development of high‐quality, reliable and publicly available patient‐directed knowledge tools with the support and acceptance of professional associations (and alliances), scientific associations and patients’ organizations. The main stakeholders (the Dutch Federation of Patients’ Organisations, the Dutch Association of Medical Specialists, the Dutch College of General Practitioners and the Dutch Nurses’ Association) continued in working together to translate the guidance into a web‐based practical version, and to arrive at consensus on a sustainable model for the development, publication, governance and financing of patient decision aids. An important follow‐up step is to crosslink this guidance to the guidance for developers of clinical practice guidelines.^1^

Formal steps towards accreditation have not yet been taken, the question being whether this procedure is needed and is warranted given the current level of evidence. In December 2016, the USA National Quality Forum released national standards for the certification of patient decision aids.^30^ The certification criteria are meant to be used for “complete” patient decision aids, which are standalone, independent tools for patients facing a clinical decision. Our guidance, although not formulated along the lines of certification criteria, is highly comparable with the USA criteria in terms of content. The only USA criterion that we do not cover is that the patient decision aid should report readability levels.

We believe that this study can be seen as an inspirational example for other countries that are facing the same challenges with regard to the development and governance of clinician‐directed and patient‐directed knowledge tools such as guidelines, guideline summaries, patient versions of guidelines and patient decision aids.

## CONFLICT OF INTEREST

All authors are involved in the development of clinical practice guidelines, none of them has any commercial interest, and they all stem from non‐profit organizations.
