# Supplementary material for: Developing quality criteria for patient‐directed knowledge tools related to clinical practice guidelines. A development and consensus study
Source: Health Expect. 2018 Nov 11;22(2):201–8. doi: 10.1111/hex.12843 (PMC6433309; doi:10.1111/hex.12843)
Supplement: Supplementary file 1 [file HEX-22-201-s001.docx]

**Developing quality criteria for patient-directed knowledge tools related to clinical practice guidelines. A systematic, inclusive approach.**

**Appendix**

**References for Patient versions of guidelines**

1. de Wit MP, Smolen JS, Gossec L, van der Heijde DM. Treating rheumatoid arthritis to target: the patient version of the international recommendations. Ann Rheum Dis. 2011 Jun;70(6):891-5.
2. Stoffer MA, Smolen JS, Woolf A, et al. Development of patient-centred standards of care for osteoarthritis in Europe: the eumusc.net-project. Ann Rheum Dis. 2015 Jun;74(6):1145-9.
3. Williams NH, Amoakwa E, Burton K, et al. The Hip and Knee Book: developing an active management booklet for hip and knee osteoarthritis. Br J Gen Pract. 2010 Feb;60(571):64-82.
4. Kiltz U, van der Heijde D, Mielants H, Feldtkeller E, Braun J; PARE/EULAR patient initiative group. ASAS/EULAR recommendations for the management of ankylosing spondylitis: the patient version. Ann Rheum Dis. 2009 Sep;68(9):1381-6.

**References for Patient Decision aids**

1. Hoffman AS, Llewellyn-Thomas HA, Tosteson AN, O'Connor AM, Volk RJ, Tomek IM, et al. Launching a virtual decision lab: development and field-testing of a web-based patient decision support research platform. BMC medical informatics and decision making. 2014;14:112.
2. Carroll SL, McGillion M, Stacey D, Healey JS, Browne G, Arthur HM, et al. Development and feasibility testing of decision support for patients who are candidates for a prophylactic implantable defibrillator: a study protocol for a pilot randomized controlled trial. Trials. 2013;14:346.
3. Fleisher L, Ruggieri DG, Miller SM, Manne S, Albrecht T, Buzaglo J, et al. Application of best practice approaches for designing decision support tools: the preparatory education about clinical trials (PRE-ACT) study. Patient education and counseling. 2014;96(1):63-71.
4. Cranney A, O'Connor AM, Jacobsen MJ, Tugwell P, Adachi JD, Ooi DS, et al. Development and pilot testing of a decision aid for postmenopausal women with osteoporosis. Patient education and counseling. 2002;47(3):245-55.
5. Elwyn G, Kreuwel I, Durand MA, Sivell S, Joseph-Williams N, Evans R, et al. How to develop web-based decision support interventions for patients: a process map. Patient education and counseling. 2011;82(2):260-5.
6. Montori VM, Breslin M, Maleska M, Weymiller AJ. Creating a conversation: insights from the development of a decision aid. PLoS Med. 2007;4(8):e233.
7. Harwood R, Douglas C, Clark D. Decision aids for breast and nodal surgery in patients with early breast cancer: development and a pilot study. Asia-Pacific journal of clinical oncology. 2011;7(2):114-22.
8. Johnson SL, Kim YM, Church K. Towards client-centered counseling: development and testing of the WHO Decision-Making Tool. Patient education and counseling. 2010;81(3):355-61.
9. Raats CJI, van Veenendaal H, Versluijs MM, Burgers JS. A generic tool for development of decision aids based on clinical practice guidelines. Patient education and counseling. 2008;73(3):413-7.
10. Wong J, D'Alimonte L, Angus J, Paszat L, Metcalfe K, Whelan T, et al. Development of patients' decision aid for older women with stage I breast cancer considering radiotherapy after lumpectomy. International journal of radiation oncology, biology, physics. 2012;84(1):30-8.
11. LeBlanc A, Bodde AE, Branda ME, Yost KJ, Herrin J, Williams MD, et al. Translating comparative effectiveness of depression medications into practice by comparing the depression medication choice decision aid to usual care: study protocol for a randomized controlled trial. Trials. 2013;14:127.
12. Ng CJ, Mathers N, Bradley A, Colwell B. A 'combined framework' approach to developing a patient decision aid: the PANDAs model. BMC health services research. 2014;14:503.
13. Ozanne EM, Howe R, Omer Z, Esserman LJ. Development of a personalized decision aid for breast cancer risk reduction and management. BMC medical informatics and decision making. 2014;14:4.
14. Stacey D, O'Connor AM, DeGrasse C, Verma S. Development and evaluation of a breast cancer prevention decision aid for higher-risk women. Health expectations : an international journal of public participation in health care and health policy. 2003;6(1):3-18.
15. Schoorel EN, Vankan E, Scheepers HC, Augustijn BC, Dirksen CD, de Koning M, et al. Involving women in personalised decision-making on mode of delivery after caesarean section: the development and pilot testing of a patient decision aid. BJOG : an international journal of obstetrics and gynaecology. 2014;121(2):202-9.
16. Breslin M, Mullan RJ, Montori VM. The design of a decision aid about diabetes medications for use during the consultation with patients with type 2 diabetes. Patient education and counseling. 2008;73(3):465-72.
17. Fleisher L, Buzaglo J, Collins M, Millard J, Miller SM, Egleston BL, et al. Using health communication best practices to develop a web-based provider-patient communication aid: the CONNECT study. Patient education and counseling. 2008;71(3):378-87.
18. Lalonde L, O'Connor AM, Drake E, Duguay P, Lowensteyn I, Grover SA. Development and preliminary testing of a patient decision aid to assist pharmaceutical care in the prevention of cardiovascular disease. Pharmacotherapy. 2004;24(7):909-22.
19. Ameling JM, Auguste P, Ephraim PL, Lewis-Boyer L, DePasquale N, Greer RC, et al. Development of a decision aid to inform patients' and families' renal replacement therapy selection decisions. BMC medical informatics and decision making. 2012;12:140.
20. Schonberg MA, Hamel MB, Davis RB, Griggs MC, Wee CC, Fagerlin A, et al. Development and evaluation of a decision aid on mammography screening for women 75 years and older. JAMA internal medicine. 2014;174(3):417-24.
21. Bansback N, Li LC, Lynd L, Bryan S. Development and preliminary user testing of the DCIDA (Dynamic computer interactive decision application) for 'nudging' patients towards high quality decisions. BMC medical informatics and decision making. 2014;14:62.
22. Warner DO, LeBlanc A, Kadimpati S, Vickers KS, Shi Y, Montori VM. Decision Aid for Cigarette Smokers Scheduled for Elective Surgery. Anesthesiology. 2015;123(1):18-28.
23. Coulter A, Stilwell D, Kryworuchko J, Mullen PD, Ng CJ, van der Weijden T. A systematic development process for patient decision aids. BMC medical informatics and decision making. 2013;13 Suppl 2:S2.
24. Stacey D, Légaré F, Col NF, Bennett CL, Barry MJ, Eden KB, et al. Decision aids for people facing health treatment or screening decisions. Cochrane Database Syst Rev. 2014;CD001431.
25. Izquierdo F, Gracia J, Guerra M, Blasco JA, Andradas E. Health technology assessment-based development of a Spanish breast cancer patient decision aid. International journal of technology assessment in health care. 2011;27(4):363-8.
26. Sherman KA, Harcourt DM, Lam TC, Shaw LK, Boyages J. BRECONDA: development and acceptability of an interactive decisional support tool for women considering breast reconstruction. Psycho-oncology. 2014;23(7):835-8.
27. Elwyn G, O'Connor A, Stacey D, Volk R, Edwards A, Coulter A, et al. Developing a quality criteria framework for patient decision aids: online international Delphi consensus process. Bmj. 2006;333(7565):417.
28. Holmes-Rovner M, Stableford S, Fagerlin A, Wei JT, Dunn RL, Ohene-Frempong J, et al. Evidence-based patient choice: a prostate cancer decision aid in plain language. BMC medical informatics and decision making. 2005;5:16.
29. Weyand SA, Frize M, Bariciak E, Dunn S. Development and usability testing of a parent decision support tool for the neonatal intensive care unit. Conference proceedings : Annual International Conference of the IEEE Engineering in Medicine and Biology Society IEEE Engineering in Medicine and Biology Society Annual Conference. 2011:6430-3.
30. Kelly-Blake K, Clark S, Dontje K, Olomu A, Henry RC, Rovner DR, et al. Refining a brief decision aid in stable CAD: cognitive interviews. BMC medical informatics and decision making. 2014;14:10.
31. Feldman-Stewart D, Brundage MD. Challenges for designing and implementing decision aids. Patient education and counseling. 2004;54(3):265-73.
32. Evans R, Elwyn G, Edwards A, Watson E, Austoker J, Grol R. Toward a model for field-testing patient decision-support technologies: a qualitative field-testing study. Journal of medical Internet research. 2007;9(3):e21.
33. Fraenkel L, Street RL, Jr., Fried TR. Development of a tool to improve the quality of decision making in atrial fibrillation. BMC medical informatics and decision making. 2011;11:59.
34. Milne J, Gafni A, Lu D, Wood S, Sauve R, Ross S. Developing and pre-testing a decision board to facilitate informed choice about delivery approach in uncomplicated pregnancy. BMC pregnancy and childbirth. 2009;9:50.
